# Genetic Assembly of Double‐Layered Fluorescent Protein Nanoparticles for Cancer Targeting and Imaging

**DOI:** 10.1002/advs.201600471

**Published:** 2017-02-17

**Authors:** Seong‐Eun Kim, Sung Duk Jo, Koo Chul Kwon, You‐Yeon Won, Jeewon Lee

**Affiliations:** ^1^ Department of Chemical and Biological EngineeringKorea UniversitySeoul02841Republic of Korea; ^2^ School of Chemical Engineering and Purdue University Center for Cancer ResearchPurdue UniversityWest LafayetteIN47906USA; ^3^ Center for TheragnosisKorea Institute of Science and TechnologySeoul02792Republic of Korea

**Keywords:** cancer targeting and imaging, double‐layered fluorescent proteins, genetic encapsulation, super‐fluorescent protein nanoparticles, viral capsid

## Abstract

Hepatitis B virus capsid (HBVC), a self‐assembled protein nanoparticle comprised of 180 or 240 subunit proteins, is used as a cage for genetic encapsulation of fluorescent proteins (FPs). The self‐quenching of FPs is controlled by varying the spacing between FPs within the capsid structure. Double‐layered FP nanoparticle possessing cancer cell‐targeting capabilities is also produced by additionally attaching FPs and cancer cell receptor‐binding peptides (affibodies) to the outer surface of the capsid. The generically modified HBVC with double layers of mCardinal FPs and affibodies (mC‐DL‐HBVC) exhibit a high fluorescence intensity and a strong photostability, and is efficiently internalized by cancer cells and significantly stable against intracellular degradation. The mC‐DL‐HBVC effectively detects tumor in live mice with enhanced tumor targeting and imaging efficiency with far less accumulation in the liver, compared to a conventional fluorescent dye, Cy5.5. This suggests the great potential of mC‐DL‐HBVC as a promising contrast agent for in vivo tumor fluorescence imaging.

## 1 Introduction

Real‐time image‐guided surgery recently has attracted considerable attention in surgical cancer treatment,^1^ which depends on intraoperative imaging techniques to guide the surgical procedure. For intraoperative imaging, fluorescence techniques that can visualize specific cancer tissues using fluorescent contrast agents are in general better suited than other nonoptical imaging techniques such as magnetic resonance imaging (MRI), computed tomography (CT), and positron emission tomography (PET).^2^ The conventional near infrared (NIR) fluorescent probes, such as Q‐dots (CdSe, ZnS, etc.) and synthetic organic dyes (Cy5, Cy5.5, etc.), fall short for this application because of their in vivo toxicity and undesirable chemical properties including nonspecific binding to plasma proteins such as albumin.^3^ One approach that has been taken to solve these problems is to encapsulate fluorescent probes within liposomes^4^ or hollow silica spheres,^5^ although they are not safe enough to solve the underlying issues of toxicity due to the possibility of long‐term accumulation in the body. These synthetic cages with reasonably stable structure^4^, ^5^ are easy to practice chemical or physical encapsulation procedures but are typically polydisperse particles with sizes ranging from a few to hundreds of nanometers, which in turn causes variation in the number of loaded fluorophore molecules per particle.^6^ Uncontrolled release of encapsulated fluorophores is another typical problem.^6^ None of the current methods provides precise control of the spatial distribution of the caged fluorophores, which makes it difficult to control the self‐quenching effect.^7^ Furthermore, hydrophilic fluorophores (e.g., fluorescent proteins) are typically net electrically charged, and therefore, encapsulating them inside small cage structures at high concentrations is difficult because of the repulsive electrostatic interactions.^8^

In the present study, we developed a new method of fluorophore encapsulation that overcomes these limitations of conventional methods. This method uses a biological protein nanoparticle (hepatitis B virus capsid, HBVC) as a nanostructure cage of uniform size and composition. The HBVC nanoparticles are produced through precisely controlled self‐assembly processes occurring inside cells. There have been several reports of viral capsids that are chemically conjugated with fluorescent labels for medical imaging applications. But the yield of chemical conjugation varies depending on the chemical structure of the fluorescent dye even if the same conjugation chemistry is used. For instance, the cowpea mosaic virus (CPMV) capsid has been chemically conjugated with A555 (Alexa Fluor 555) and A488 (Alexa Fluor 488) at different dye‐to‐capsid molar ratios, 70 and 120, respectively.^9^ A different study reported that the maximum number of fluorophores (fluorescein) attached to total 300 subunits comprising the CPMV capsid were limited to 200.^10^ Here we developed the fluorescent viral capsids (HBVCs) through the genetic conjugation of fluorescent protein (FP) (mCardinal or eGFP) to the individual capsid subunit, thereby resulting in the constant ratio of FP to capsid irrespective of the type of FP.

Recently, a far‐red fluorescent protein having a strong fluorescence and a high photostability, called mCardinal, has been developed:^11^ it has a peak excitation within the optical wavelength range (600–1000 nm) appropriate for deep tissue imaging.^12^ Though promising, mCardinal has been applied to in vitro imaging or in vivo implantable cells,^11^, ^13^ but not yet applied to in vivo tumor imaging. In the present study, mCardinal FPs were genetically conjugated to the inner and outer surfaces of HBVC, which gives rise to double‐layered mCardinal HBVC nanoparticle (mC‐DL‐HBVC) possessing an enhanced fluorescence and photostability. Here we report detailed structural, optical, in vitro and in vivo tumor cell targeting and imaging, and in vivo biodistribution properties of mC‐DL‐HBVC. The results demonstrate that mC‐DL‐HBVC has a great potential as a contrast agent for in vivo tumor fluorescence imaging.

## 2 Results and Discussion

### 2.1 Genetic Modification of HBVC for Assembly and Encapsulation of Fluorescent Proteins

**Scheme 1:**
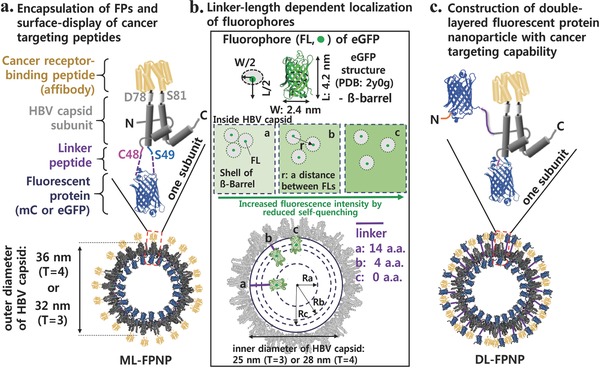
Genetic encapsulation and assembly to produce fluorescently engineered HBV capsids with cancer targeting capability. a) Encapsulation of FPs surface‐display of cancer targeting peptides. b) Linker‐length dependent localization of fluorophores. c) Construction of double‐layered fluorescent protein nanoparticle with cancer targeting capability.

As shown in **Scheme** **1**, the HBVC subunits^14^ that are genetically conjugated to FPs (enhanced green fluorescent protein/eGFP or mCardinal/mC) were self‐assembled to mono‐ or double‐layered FP nanoparticles (ML‐ or DL‐FPNPs, respectively) in the cytoplasm of *Escherichia coli*. The genetic insertion of FP into a particular site between Cys48 and Ser49 of HBVC subunit leads to the formation of internal layer of FPs upon the self‐assembly of the modified subunits. The length of the linker peptide between the FP and HBVC subunit was varied to examine its influence on the fluorescence intensity of FPNPs. We found that the fluorescence intensity reached maximum when no peptide linker was used, which is probably because the separation between adjacent FPs in the capsid interior is maximized, and thus the self‐quenching effect becomes minimized (Scheme 1a,b). The additional genetic insertion of FP to the N‐terminus of capsid subunit resulted in the synthesis of DL‐FPNPs having significantly enhanced fluorescence intensity and photostability compared to the native FPs (Scheme 1c). Further, the cancer cell receptor‐binding peptides, a tandem repeat of affibody peptide with specific and strong affinity for human epidermal growth factor receptor I (EGFR) that is overexpressed on the surface of many types of cancer cells,[[qv: 14b,15]] were genetically presented on the outer surface of the DL‐FPNP (Scheme 1a,c), enabling the DL‐FPNP to have dual modality of cancer cell targeting and imaging.

### 2.2 Structural and Optical Properties of Genetically Encapsulated Fluorescent Protein Nanoparticles

**Figure 1:**
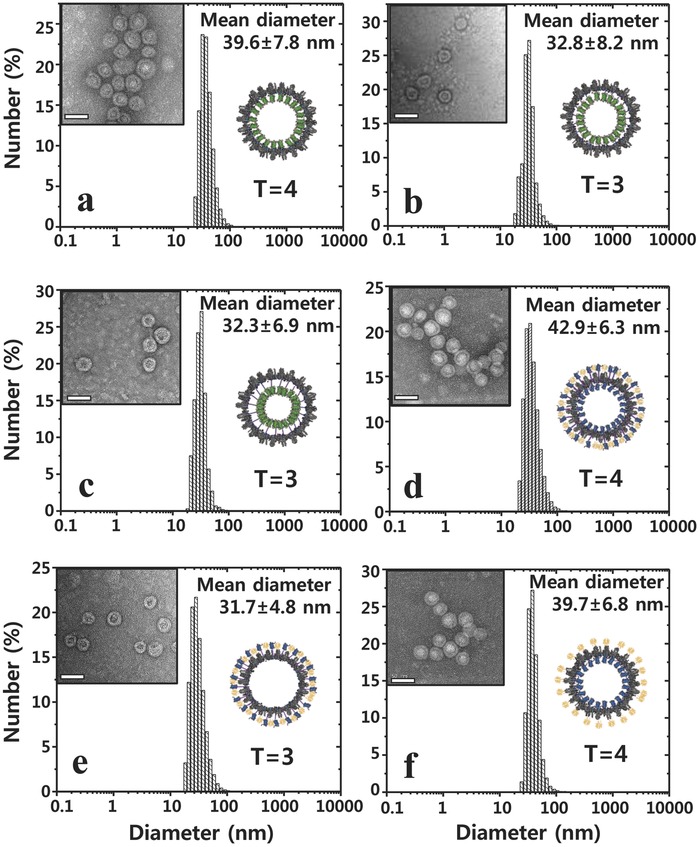
TEM images and size distributions (determined by DLS) of purified eGFP_in_‐HBVC with different linker lengths [*n* = a) 0, b) 4, and c) 14], d) mC‐DL‐HBVC, e) mC_out_‐HBVC, and f) mC_in_‐HBVC. Scale bars represent 50 nm. All of eGFP_in_‐HBVC, mC‐DL‐HBVC, mC_out_‐HBVC, and mC_in_‐HBVC present affibody peptides on their outer surface, as described in Scheme 1a,c.

First, eGFP was used as a model FP, because its structure and fluorescence characteristics (e.g., Förster distance, *R*_F_ = 4.65 nm) have been well characterized.^16^ Three different linker peptides were used for connecting the eGFP to the capsid subunit (number of amino acids in the linker peptide (*n*) = 0, 4, and 14) (Scheme 1b). Flexible glycine‐rich linkers were used in order to minimize steric interference between the eGFP and capsid subunit (Figure S1, Supporting Information). Transmission electron microscope (TEM) shows that the modified capsid subunits by inserting eGFP into the site between Cys48 and Ser49 (Scheme 1a) were self‐assembled to spherical protein nanoparticles (named eGFP_in_‐HBVCs) in *E. coli* (**Figure** **1**a–c). Western blot analysis confirmed that eGFPs were successfully localized inside HBVC (Figure S2, Supporting Information). In wild‐type HBVC, two different types of icosahedral capsids have been observed: *T* = 3 (32 nm) and *T* = 4 (36 nm) symmetry‐based capsids consisting of 180 and 240 subunits, respectively.^17^ In this work, we used the truncated mutant (1–149 a.a.) of HBVC subunit, which reportedly prefers the *T* = 4 symmetry upon capsid formation.^18^ Interestingly, it is notable that the assembly symmetry of eGFP_in_‐HBVCs depended on the linker length: when *n* = 0, the capsid with *T* = 4 symmetry was formed, but when *n* = 4 or 14, the capsid takes smaller structures with *T* = 3 symmetry (**Table** **1** and Figure 1a–c), which indicates that depending on genetic modification of interior space of the capsid, the capsid subunits are self‐assembled through different symmetry to form differently sized nanoparticles, as reported previously.^19^

**Table 1:** Physicochemical properties of fluorescently engineered HBV capsids

Type of fluorescently engineered HBVC	MW of modified capsid subunita) [Da]	Diameterb) [nm]	Capsid symmetry	Zeta potential [mV]
eGFP_in_‐HBVC (*n* = 0)	49 293.7	39.6 ± 7.8	*T* = 4	−5.6 ± 0.4
eGFP_in_‐HBVC (*n* = 4)	49 881.3	32.8 ± 8.2	*T* = 3	−6.2 ± 0.5
eGFP_in_‐HBVC (*n* = 14)	51 170.5	32.3 ± 6.9	*T* = 3	−5.6 ± 0.7
mC_in_‐HBVC (*n* = 0)	64 847.3	39.7 ± 6.8	*T* = 4	−2.4 ± 1.0
mC_out_‐HBVC (*n* = 0)	64 847.3	31.7 ± 4.8	*T* = 3	−5.4 ± 0.5
mC‐DL‐HBVC (*n* = 0)	92 264.5	42.9 ± 6.3	*T* = 4	−5.7 ± 0.3

^a)^ Calculated using Expasy.org

^b)^ Measured by DLS.

**Figure 2:**
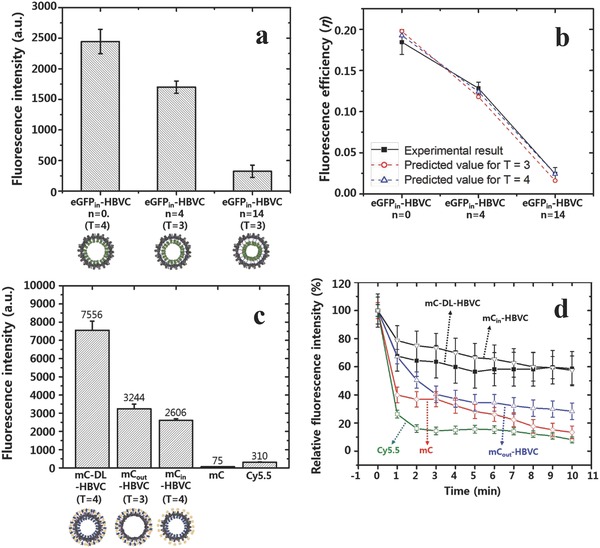
a) Fluorescence intensities of eGFP_in_‐HBVCs with different linker lengths (*n* = 0, 4, and 14) at an identical molar concentration of eGFP (2 × 10^−9^ m). b) Comparison of the experimentally determined and theoretically calculated fluorescence efficiencies of eGFP_in_‐HBVCs with different linker lengths (*n* = 0, 4, and 14) and assembly symmetries (*T* = 3 and 4). c) Fluorescent intensities of mC_in_‐HBVC, mC_out_‐HBVC, mC‐DL‐HBVC, and pristine mC at an identical molar concentration of mC (2 × 10^−9^ m) and Cy5.5 (2 × 10^−9^ m). d) Photostability of mC_in_‐HBVC, mC_out_‐HBVC, mC‐DL‐HBVC, pristine mC, and Cy5.5 under continuous NIR irradiation. Values represent means of triplicates with standard deviations shown as error bars.

As shown in **Figure** **2**a, the fluorescence signals of eGFP_in_‐HBVCs significantly decreased as the linker peptide length increased from *n* = 0 to 14, which is probably due to the reduced distance (*r*) between fluorophores of two adjacent eGFPs and accordingly due to the increased self‐quenching effect in the capsid interior. The correlation between linker length and self‐quenching effect was also analyzed through theoretical calculation of fluorescence efficiency (η) using fluorescence energy transfer equation (Equation (1) of the Experimental Section)^16^ and known molecular geometric parameters for eGFP (Table S1, Supporting Information). The predicted values of η were calculated for the three different linker lengths (*n* = 0, 4, and 14) and for the two different symmetries (*T* = 3 and 4) and then compared with the experimentally estimated values using the equation, η = QY × (1 – *E*), where QY and *E* denote the quantum yield and energy transfer efficiency of eGFP, respectively. *E* was estimated from the measured fluorescence intensities of eGFP_in_‐HBVC (*F*) and pristine eGFPs (*F*_o_) using the equation, *E* = 1 – *F*/*F*_o_. From Figure 2b and **Table** **2**, the predicted η showed excellent agreement with the experimental values, while it was not so sensitive to the capsid symmetry, supporting that the linker length is indeed a key parameter that controls the self‐quenching effect.

**Table 2:** Comparison of the experimentally determined and theoretically calculated fluorescence efficiencies (η) of eGFP_in_‐HBVCs with the different linker lengths

Linker length of eGFP_in_‐HBVC	η from experimental measurementsa)	η from theoretical calculation for *T* = 3b)	η from theoretical calculation for *T* = 4b)
*n* = 0	0.184 ± 0.015	0.200	0.190
*n* = 4	0.128 ± 0.001	0.120	0.120
*n* = 14	0.024 ± 0.001	0.016	0.024

^a)^ Estimated from the measured fluorescence intensities (*F* and *F*_o_ in the equation, η = QY(*F*/*F*_o_)

^b)^ Calculated using the equation, η = QY[*r* ^6^/(*r* ^6^ + *R*_Q_^6^)]. (For eGFP, QY = 0.6, and *R*_Q_ = 3.49; see the Supporting Information for details.)

### 2.3 Structural and Optical Properties of Genetically Assembled and Mono‐ or Double‐Layered mCardinal Protein Nanoparticles

As presented in Scheme 1, mC was also genetically conjugated to the capsid subunit to form mono‐ (mC_out_‐ and mC_in_‐HBVCs) and double‐layered (mC‐DL‐HBVC) fluorescent protein nanoparticles. mC is a recently developed FP that emits fluorescence when irradiated by a deeper tissue‐penetrating NIR light.^11^ Based on the results of Figure 2a,b, mC was inserted to the site between Cys48 and Ser49 of capsid subunit without any linker peptide (*n* = 0) in the synthesis of mC_in_‐HBVC. To produce mC_out_‐HBVC and mC‐DL‐HBVC, mC was additionally conjugated to the N‐terminus of capsid subunit using a glycine‐rich flexible linker with about 12 nm length (Figure S1, Supporting Information; Scheme 1). As described in Scheme 1a,c, mC‐DL‐HBVC, mC_out_‐HBVC, and mC_in_‐HBVC that have all spherical nanoparticle shape present cancer cell receptor (EGFR)‐binding affibody peptides on their outer surface (Figure 1d–f; Table 1). Interestingly, like the linker‐free eGFP_in_‐HBVCs (Figure 1a), the assembly symmetry of mC_in_‐HBVC and mC‐DL‐HBVC is *T* = 4 (Figure 1d,f), while mC_out_‐HBVC showed *T* = 3 symmetry (Figure 1e). This indicates that the linker‐free insertion of FP into the site between Cys48 and Ser49 of capsid subunit always leads to *T* = 4 assembly irrespective of genetic modification of N‐terminus and loop region of capsid subunit. All these mC‐containing HBVC nanoparticles exhibited negative zeta potentials (Table 1).

As shown in Figure 2c, the mC‐DL‐HBVC emits the highest‐level fluorescence among the tested fluorophores. It is noticeable that the fluorescence intensity of mC‐DL‐HBVC was about 23% higher than the sum of the intensities from mC_out_‐ and mC_in_‐HBVCs. This is due to the fact that the mC_out_‐HBVC (*T* = 3 symmetry, 32 nm) consists of lower number (180) of subunits than the mC‐DL‐HBVC and mC_in_‐HBVC (*T* = 4 symmetry) with 240 subunits (Table 1). (If the fluorescence intensity of mC_out_‐HBVC is proportionally increased according as the symmetry of mC_out_‐HBVC is assumed to be *T* = 4, the sum of the fluorescence intensities from mC_out_‐ and mC_in_‐HBVCs is exactly equal to that of mC‐DL‐HBVC.) Notably, the mC‐DL‐HBVC exhibited a 24‐fold higher fluorescence intensity than Cy5.5 at an identical molar concentration (Figure 2c). The photostability was also examined by measuring time‐course change of fluorescence intensity under continuous irradiation of NIR light. As shown in Figure 2d and Figure S3 (Supporting Information), mC monomer and mC_out_‐HBVC lost 70%–80% of its initial fluorescence intensity within 10 min, whereas both mC_in_‐HBVC and mC‐DL‐HBVC retained about 60% of their initial fluorescence intensity even after 10 min. Cy5.5 was least photostable among all tested. These results clearly indicate that the HBVC is a robust biological cage that effectively protects the encapsulated mCs against photobleaching.

### 2.4 In Vitro and In Vivo Tumor Targeting and Imaging Using Double‐Layered mCardinal Protein Nanoparticles as a Fluorescent Contrast Agent

**Figure 3:**
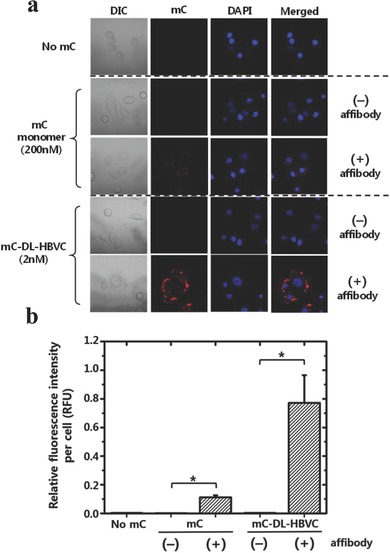
Cellular uptake of mC‐DL‐HBVC (2 × 10^−9^ m) and mC monomer (200 × 10^−9^ m) with and without affibody peptides by EGFR‐overexpressing cancer cells (MDA‐MB‐468) in vitro at 37 °C. a) Confocal microscopy images of MDA‐MB‐468 cells. Nuclei were counterstained with DAPI (blue). b) Fluorescence intensities from the MDA‐MB‐468 cells of (a). Error bars represent standard deviations, *n* ≥ 7. *: *p*‐value < 0.05.

**Figure** **3**a,b shows that in the absence of the EGFR affibody, neither mC‐DL‐HBVC nor the mC monomer was internalized by MDA‐MB‐468 cells, indicating that the surface‐exposed affibody peptides effectively mediated the cellular uptake process. Notably, mC‐DL‐HBVC at the 100‐times lower concentration produces a sevenfold higher fluorescence signal from the cancer cells than the mC monomer (Figure 3b). The cytotoxicity of mC‐DL‐HBVC was also evaluated in vitro using the Cell Counting Kit‐8 (CCK‐8) method. Cancer cells (MDA‐MB‐468) treated with mC‐DL‐HBVC showed no indication of toxicity over all concentrations tested (Figure S4, Supporting Information).

**Figure 4:**
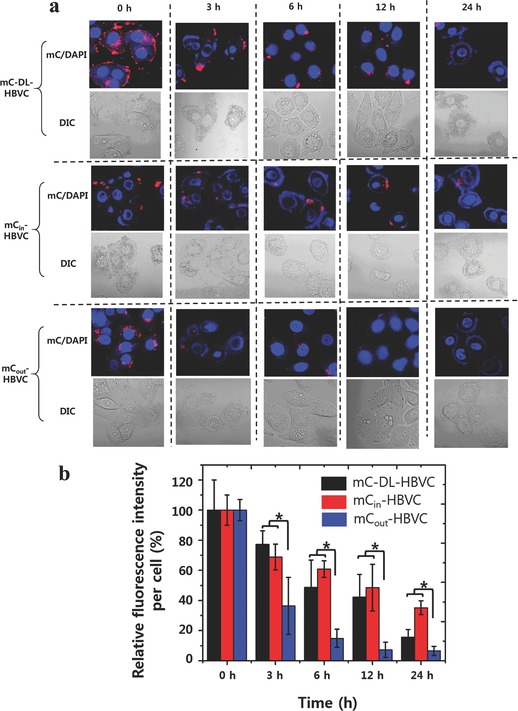
Time‐course analysis of the intracellular fluorescence of MDA‐MB‐468 cancer cells treated with mC‐DL‐HBVC, mC_in_‐HBVC, and mC_out_‐HBVC, both of which present affibody peptides on their surface. a) Confocal microscopy images of MDA‐MB‐468 cells at predetermined time points after treated with mC‐DL‐HBVC and mC_out_‐HBVC. Nuclei were stained with DAPI (blue). b) Time‐course fluorescence intensities from the MDA‐MB‐468 cells of (a). At each time point, the fluorescence intensities were normalized by the respective initial value. Error bars represent standard deviations, *n* ≥ 7. *: *p*‐value < 0.05.

MDA‐MB‐468 cells were first treated with mC‐DL‐HBVC (2 × 10^−9^ m), mC_in_‐HBVC (2 × 10^−9^ m), or mC_out_‐HBVC (2 × 10^−9^ m) for 3 h, washed to remove uninternalized HBVCs, and incubated with fresh medium for additional 24 h at 37 °C. At the predetermined time points (3, 6, 12, and 24 h), the fluorescence signals of cancer cells were monitored/recorded using a confocal microscope (**Figure** **4**a). The mC‐DL‐ and mC_in_‐HBVC‐treated cells retained more than 40% of the initial fluorescence signal at 12 h, whereas the mC_out_‐HBVC‐treated cells lost more than 90% of the original intensity within 12 h (Figure 4b), indicating that the HBV capsid provides a protection of the encapsulated mC fluorophores against degradation in the intracellular environment for a prolonged period of time. According to the previous literature,^20^ the intracellular localization of fluorescent molecules can be determined from fluorescent cell images: punctate fluorescence patterns typically imply the confinement of fluorescent molecules within endosomal compartments, whereas smeared/diffuse fluorescent signals indicate the signaling molecules in cytosol. Figure 4a clearly shows the punctate fluorescence patterns in cells, suggesting that the intracellular mC‐DL‐HBVC, mC_out_‐HBVC, and mC_in_‐HBVC are located inside endosomes (or lysosomes) at the time of imaging. This is well supported by Figure 4b, showing that the fastest decrease of fluorescent intensity was observed in tumor cells treated with mC_out_‐HBVC, while the slowest was with mC_in_‐HBVC. It seems that the low‐pH‐degradable mCardinal^11^ was effectively protected when it was encapsulated within the HBVC that is known to be stable over a wide range of pH (4–10).^21^

**Figure 5:**
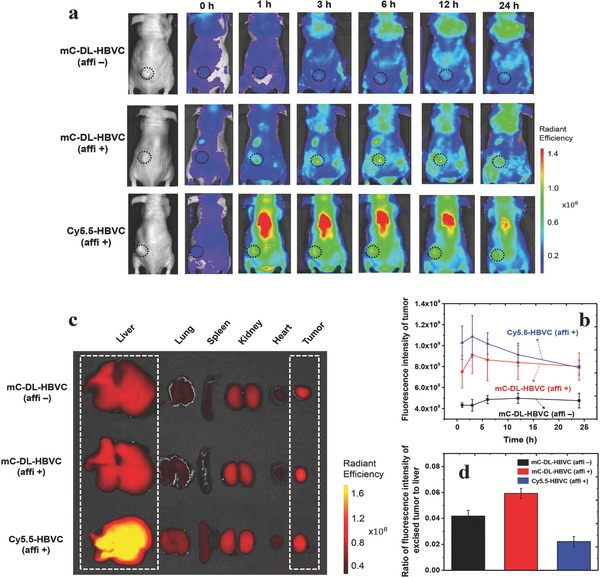
a) Time‐course NIR fluorescence images of MDA‐MB‐468 tumor‐bearing mice after the intravenous injection of mC‐DL‐HBVC (affi +), mC‐DL‐HBVC (affi −), and Cy5.5‐HBVC (affi +). b) Time‐course variation in the fluorescence intensity of tumor in MDA‐MB‐468 or A431 tumor‐bearing mice injected with the same fluorophores of (a). c) Ex vivo NIR fluorescence images of five organs (liver, lung, spleen, kidney, and heart) and tumor that were excised from MDA‐MB‐468 tumor‐bearing mice at 24 h postinjection of the same fluorophores of (a). d) Fluorescence intensity ratio of the excised tumor to liver from MDA‐MB‐468 or A431 tumor‐bearing mice injected with the same fluorophores of (a). Error bars represent standard deviations (*n* = 4).

The mC‐DL‐HBVCs with and without affibody peptides (affi + and affi −, respectively) were intravenously injected to EGFR‐overexpressing tumor (MDA‐MB‐468 or A431)‐bearing mice (*n* = 4), and in vivo distribution and delivery of the mC‐DL‐HBVCs to the tumor were monitored at predetermined time points for 24 h using fluorescence optical imaging system. A conventional NIR dye (Cy5.5)‐labeled HBVC with affibody peptides (Cy5.5‐HBVC (affi +)) was also injected to the same tumor‐bearing mice. Prior to the injection to live mice, the fluorescence intensity of mC‐DL‐HBVC (affi +), mC‐DL‐HBVC (affi −), and Cy5.5‐HBVC (affi +) was all equally adjusted (see the Experimental Section). From **Figure** **5**a, the mC‐DL‐HBVC (affi +) clearly detected the tumor with the highest image contrast in NIR fluorescence image of whole‐body mice. From Figure 5b, it is obvious that the affibody peptides significantly enhanced the tumor targeting and detection by mC‐DL‐HBVC, but the highest fluorescence intensity of tumor was observed in the tumor‐bearing mice injected with Cy5.5‐HBVC (affi +). The analysis of ex vivo fluorescence images of excised tumor and five major organs demonstrates that much larger amount of Cy5.5‐HBVC (affi +) accumulated in liver compared to the mC‐DL‐HBVCs (Figure 5c), leading to the lowest level in the ratio of tumor to liver fluorescence intensity in the injection of Cy5.5‐HBVC (affi +) (Figure 5d). Reportedly, Cy5.5 has a high tendency of accumulation in liver due to its lipophilicity and negative charge.[[qv: 3b,22]] (The measured zeta potential of Cy5.5‐HBVC (affi +) was – 8.45 mV.) The mC‐DL‐HBVC (affi +) that is a protein‐based hydrophilic fluorescent molecule shows much less accumulation in liver as well as higher fluorescence intensity and photostability and significantly better performance in in vivo tumor imaging than conventional organic dyes like Cy5.5. Compared to other fluorescent proteins producing shorter wavelength emissions (e.g., mRuby3 and mScarlet),^23^ mC‐DL‐HBVC would perhaps not produce brighter signals in vivo. However, mC‐DL‐HBVC has clear advantages in terms of photostability and longer emission wavelength. Therefore, we believe that mC‐DL‐HBVC is well suited for real‐time image‐guided surgery that requires a photostable imaging reagent with longer emission wavelength.

## 3 Conclusion

FPs were genetically encapsulated within the HBVC by inserting a FP to the site between Cys48 and Ser49 of capsid subunit, while the genetic conjugation of FP to N‐terminus of capsid subunit presented FPs on the outer surface of HBVC. Further, cancer cell receptor‐binding ligands (affibody peptides) were genetically displayed on the external surface of fluorescently engineered HBVC. The modified capsid subunits were self‐assembled to mono‐ or double‐layered FPNP with cancer targeting capability in *E. coli*. The length of linker peptide between the internally localized FP and the capsid strongly influences self‐quenching effect of the FPs inside the capsid cage: the shorter the linker is, the stronger the fluorescence signal becomes, due to reduced self‐quenching. Also, depending on the internal linker length and the insertion site of FP, the modified capsid subunits are assembled through two different symmetries, *T* = 3 for smaller FPNPs with 180 subunits and *T* = 4 for larger FPNPs with 240 subunits. In particular, the internal linker‐free mC‐DL‐HBVCs adopts *T* = 4 symmetry, producing the highest‐level fluorescence and exhibiting far enhanced photostability against photobleaching. The affibody‐presenting mC‐DL‐HBVC was effectively endocytosed by EGFR‐overexpressing tumor cells and less susceptible to intracellular degradation. Notably, the intravenously injected mC‐DL‐HBVC with affibody peptides effectively targeted and imaged tumor in live mice with much lower accumulation in liver compared to hydrophobic organic dye (Cy5.5), leading to higher tumor‐image contrast in the whole‐body imaging of tumor‐bearing mice. Although was used for proof‐of‐concept in this study, the affibody peptides can be easily switched to other tumor cell receptor‐binding peptides. Taken together, mC‐DL‐HBVC with tumor targeting capability seems to have a high potential as a promising contrast agent for in vivo fluorescent imaging of tumor.

## 4 Experimental Section

*Biosynthesis and Characterization of Fluorescently Engineered HBV Capsids (eGFP_in_‐HBVC, mC_out_‐HBVC, mC_in_‐HBVC, and mC‐DL‐HBVC)*: By polymerase chain reaction with appropriately designed primers, ten gene clones were prepared from a previously cloned expression vector for HBV capsid subunit protein,[[qv: 14a,b]] encoding the following proteins: NH_2_‐*Nde*I‐H_6_‐P1 (MASSLRQILDSQKMEWRSNAGG SG_3_SG_3_TG_6_Y_6_)‐capsid subunit (1–48)‐linker (*n* = 14: G_3_SG_3_TG_6_)‐eGFP‐linker (*n* = 14: G_3_SG_3_TG_6_)‐capsid subunit (49–149)‐*Cla*I‐COOH, NH_2_‐*Nde*I‐H_6_‐P1‐capsid subunit (1–48)‐linker(*n* = 4: G_4_)‐eGFP‐linker (*n* = 4: G_4_)‐capsid subunit (49–149)‐*Cla*I‐COOH, NH_2_‐*Nde*I‐H_6_‐P1‐capsid subunit (1–48)‐eGFP‐capsid subunit (49–149)‐*Cla*I‐COOH, NH_2_‐*Nde*I‐H_6_‐eGFP‐*Cla*I‐COOH, NH_2_‐*Nde*I‐H_6_‐P1‐capsid subunit (1–48)‐mC‐capsid subunit (49–78)‐P2 (G_3_SG_3_TG_3_SG_3_)‐Affi [(EGFR‐binding affibody peptide)_2_]‐P2‐capsid subunit (81–149)‐*Cla*I‐COOH, NH_2_‐*Nde*I‐H_6_‐mC‐P1‐capsid subunit (1–78)‐P2‐Affi‐P2‐capsid subunit (81–149)‐*Cla*I‐COOH, NH_2_‐*Nde*I‐H_6_‐mC‐P1‐capsid subunit (1–48)‐mC‐capsid subunit (49–78)‐P2‐Affi [(EGFR‐binding affibody peptide)_2_]‐P2‐capsid subunit (81–149)‐*Cla*I‐COOH, NH_2_‐*Nde*I‐H_6_‐mC‐P1‐capsid subunit (1–48)‐mC‐capsid subunit (49–149)‐*Cla*I‐COOH, NH_2_‐*Nde*I‐H_6_‐mC‐*Cla*I‐COOH, and NH_2_‐*Nde*I‐H_6_‐mC‐P2‐Affi‐*Cla*I‐COOH. Then these genes were subcloned into pT7‐7 plasmid to construct the following expression vectors: pT7‐eGFPin‐linker14‐HBVC, pT7‐eGFPin‐linker4‐HBVC, pT7‐eGFPin‐linker0‐HBVC, pT7‐eGFP, pT7‐mCin‐HBVC‐Affi, pT7‐mCout‐HBVC‐Affi, pT7‐mC‐DL‐HBVC‐Affi, pT7‐mC‐DL‐HBVC, pT7‐mC, and pT7‐mC‐Affi, respectively (Figure S1, Supporting Information). After the DNA sequences were verified, *E. coli* Rosetta (DE3) (Novagen, Darmstadt, Germany) was transformed with each of the expression vectors above. Ampicillin‐resistant transformants were selected and grown in Luria‐Bertani media at 37 °C, induced with isopropyl β‐D‐1‐thiogalactopyranoside (IPTG) (1 × 10^−3^ m) at OD_600_ = 0.6, and further cultured for 16 h at 20 °C. Finally, the synthesized recombinant proteins or protein nanoparticles were purified using an Ni^+2^‐affinity column (Qiagen, Hilden, Germany) followed by sucrose gradient ultracentrifugation.^24^ The detailed procedures for the recombinant gene expression and purification have been described in the previous publications.[[qv: 14a]]

To verify that the eGFPs are successfully encapsulated inside eGFP_in_‐HBVC, Western blot analysis was performed subsequently after 12% native polyacrylamide gel electrophoresis (PAGE). After the native PAGE, the proteins in native gel were transferred to a polyvinylidene fluoride (PVDF) membrane for Western blot analysis. For Western blot analysis, mouse anti‐his_6_ antibody (IgG) (Gene Tex, Inc., San Antonio, TX, USA) and mouse anti‐eGFP antibody (IgG) (Sigma‐Aldrich, St. Louis, MO, USA) were used as primary antibodies, and the Horseradish peroxidase‐conjugated goat anti‐mouse antibody (Cat No. 31430, Pierce, Rockford, IL, USA) was used as secondary antibody. The detailed procedures for Western blot analysis have also been described in a previous publication.^25^

The size distribution and surface charge characteristics of the fluorescently engineered HBV capsids (eGFP_in_‐HBVC, mC_in_‐HBVC, mC_out_‐HBVC, and mC‐DL‐HBVC) were analyzed using a Zetasizer Nano ZS (Malvern Instruments, Ltd., Worcestershire, United Kingdom) equipped with a 633 nm laser, for which the solution samples were prepared at a protein concentration of 1.0 mg mL^−1^ in a storage buffer (0.3 m NaCl, 0.01 m Tris‐HCl, pH 7.5). The morphologies of the fluorescently engineered HBV capsids were examined by TEM (CM‐200 electron microscope, Philips, CA, USA) at an acceleration voltage of 80 kV. To prepare TEM specimens, a 7 μL droplet of the protein suspension (0.1 mg mL^−1^) was placed onto a carbon precoated copper grid. After 1 min of waiting time (for protein deposition onto the grid surface), the remaining liquid was removed from the grid by air drying, and the grid was washed three times with distilled water. To negatively stain the TEM specimen, 7 μL of 2% (w/v) uranyl acetate solution was placed on the grid, and then the sample was dried under air.

*Estimation of Photostabilities of Fluorescently Engineered HBV Capsids (eGFP_in_‐HBVC, mC_out_‐HBVC, mC_in_‐HBVC, and mC‐DL‐HBVC), mC, and Cy5.5*: To estimate the photostabilities of fluorescent proteins and dye, the fluorescence intensities of all the samples were initially adjusted to the same level (20 000 RFU). 10 μL of each sample (pH 7.4) was dropped onto a glass cover slip, and the fluorescence of the sample was monitored every 60 s for 10 min under continuous excitation (590–650 nm) using a fluorescence microscope (Olympus IX81, Tokyo, Japan A 75 W Xenon Apo lamp attached to the fluorescence microscope was used as the excitation source. The ImageJ software (National Institutes of Health, MD, USA) was used to quantify the fluorescence intensities from the captured fluorescence images (Figure S3, Supporting Information).

*Calculations of the Fluorescence Efficiencies (η) of the eGFP_in_‐HBVCs with Different Linker Length*: Fluorescence efficiency of fluorophores under influence of self‐quenching effect is calculated using the following equation^16^

(1) $$\eta \left(r\right)=\mathrm{QY}\left(\frac{r^{6}}{r^{6}+R_{Q}{}^{6}}\right)\;\;=\mathrm{QY}\left(1-E\right)=\mathrm{QY}\left(\frac{F}{F_{o}}\right)$$

where QY is quantum yield (= 0.6 for eGFP),^16^ *r* is distance between two fluorophores, *R*_Q_ is effective quenching radius [= (*Nε*)^1/6^ *R*_F_ = 3.49, where *N* is effective number of fluorescence acceptors in the nearest neighbor (= 6 under the assumption that fluorophores inside eGFP_in_‐HBVC are closely packed through hexagonal arrangement on the internal spherical surface), ε is loss parameter between 0 and 1 (= 0.03 in this study), and *R*_F_ is Förster distance (= 4.65 nm for eGFP)^16^]; *E* is energy transfer efficiency; and *F* and *F*_o_ are fluorescence intensity of encapsulated eGFPs in eGFP_in_‐HBVC and eGFP monomer, respectively.

*In Vitro Cancer Cell Culture and Cellular Fluorescence Imaging Assays*: Cellular uptake of mC‐DL‐HBVC by EGFR‐overexpressing MDA‐MB‐468 cells was estimated and compared with that of mC monomer. MDA‐MB‐468 cells were cultured in Leibovitz L15 (Sigma Chemical Company, St. Louis, IL, USA) containing fetal bovine serum (10%), penicillin (100 U mL^−1^), and streptomycin (100 μg mL^−1^), seeded in 35 mm coverslip bottom dishes (1 × 10^4^ cells per dish), and cultured for 36 h. After washing with phosphate buffer saline (PBS) (pH 7.4), MDA‐MB‐468 cells were incubated for 60 min in serum‐free medium containing each of four different samples, mC‐DL‐HBVC with and without affibody peptides (2 × 10^−9^ m) and mC monomer with and without affibody peptides (200 × 10^−9^ m). After washing with PBS (pH 7.4), the mC‐DL‐HBVC or mC monomer‐treated cells were fixed in 4% paraformaldehyde and 0.1% glutaraldehyde for 10 min and then counterstained with 4′,6′‐diamidino‐2‐phenylindole hydrochloride (DAPI). After washing once again with PBS, the cells in PBS were imaged using a confocal laser scanning microscope (LSM 700, Carl‐Zeiss, Jena, Germany). Fluorescence excitation and emission measurement were performed using 523–635 and 560–675 nm long pass filter, respectively. Each fluorescence image was analyzed by ImageJ software to quantity the fluorescence intensity. As described in the previous publication,[[qv: 14b,15b]] the total intensity from the region of interest (ROI) (region of interest) containing more than seven cells was calculated, and this value was divided by the number of cells to obtain the value of fluorescence intensity per cell.

For the time‐course analysis of intracellular fluorescence of MDA‐MB‐468 cells, MDA‐MB‐468 cells were first incubated with mC_out_‐HBVC or mC‐DL‐HBVC with affibody peptides (2 × 10^−9^ m) in serum‐free medium for 3 h. After washing three times with PBS, the cells were further incubated with Leibovitz L15 (Sigma Chemical Company, St. Louis, IL, USA) containing fetal bovine serum (10%), penicillin (100 U mL^−1^), and streptomycin (100 μg mL^−1^) at 37 °C. At the predetermined time points (0, 6, and 12 h), the cells were fixed in 4% paraformaldehyde and 0.1% glutaraldehyde for 10 min and stained with DAPI, and the cellular fluorescence images were obtained using a confocal laser scanning microscope (LSM 700, Carl‐Zeiss, Jena, Germany), followed by the image analysis using ImageJ software to quantify the fluorescence intensity using the same procedure as described above.

*In Vitro Cytotoxicity Assay*: The cytotoxicity of mC‐DL‐HBVC in MDA‐MB‐468 cells was evaluated using the CCK‐8 assay technique. MDA‐MB‐468 cells were seeded in a 96‐well microplate at a density of 1 × 10^4^ cells per well, and cultured for 24 h at 37 °C using the Leibovitz's L‐15 medium (Sigma Chemical Company, St. Louis, IL, USA) containing fetal bovine serum (10%), penicillin (100 U mL^−1^), and streptomycin (100 μg mL^−1^). Cells in different wells were treated with mC‐DL‐HBVC at different concentration (0 × 10^−9^, 0.25 × 10^−9^, 0.5 × 10^−9^, 1 × 10^−9^, 2 × 10^−9^, and 5 × 10^−9^ m), and incubated for additional 24 h. Finally, the mC‐DL‐HBVC‐treated cells were treated with the CCK‐8 staining solution, incubated for 2 h, and subjected to absorbance measurements (450 nm) on a plate reader. Cell viability was determined as the ratio of the number of live cells in treated groups relative to the number of live cells in untreated control groups. The results were presented as means ± standard deviations (*n* = 3).

*In Vivo and Ex Vivo Fluorescence Imaging*: All animal experiments were performed in accordance with The Korea Institute of Science and Technology (KIST) guidelines on the use of animals in research. For the estimation of in vivo fluorescence imaging of MDA‐MB‐468 or A431 tumor in live mice, EGFR‐overexpressing MDA‐MB‐468 or A431 cells (1 × 10^7^ cells per mouse) in PBS (80 μL) were subcutaneously inoculated to the left back of the BALB/c nude mice (20–25 g, Nara Biotech, Seoul, Korea) (*n* = 4). Cy5.5‐HBVC was initially prepared by following the same procedures reported in the previous literature.[[qv: 14b]] DL‐HBVC (affi +), DL‐HBVC (affi −), and Cy5.5‐HBVC (affi +) in PBS (200 μL) that have the same fluorescence intensity (20 000 RFU) were intravenously injected into MDA‐MB‐468 or A431 tumor‐bearing BALB/c mouse via a tail vein.

In vivo whole‐body imaging of mice was performed using an IVIS spectrum imaging system (Caliper Life Sciences, Hopkinton, MA) at predetermined time points. The excitation/emission channel were 600/670 nm for the DL‐HBVCs and 660/710 nm for Cy5.5‐HBVC (affi +). The analysis of collected images and quantification of fluorescence intensity were analyzed by the IVIS software, and the fluorescence intensities were normalized as radiant efficiency (photons/s/cm^2^/sr per µW/cm^2^). For the quantitative analysis of fluorescence intensity of tumor, a constant area of the ROI was drawn over tumor, and the average fluorescence intensity for each tumor area was measured and presented as means ± standard deviation (*n* = 4). In the photographic images of tumor‐bearing mice (left panels of Figure 5a), the ROIs were defined as the region containing the subcutaneous tumor xenografts as indicated by dashed circles. For each mouse, the same ROI definition was used to estimate the time‐dependent variation in the fluorescence intensity from the tumor. (The same procedure has also been demonstrated in the previous publication.[[qv: 14b,15b]])

After the in vivo NIR fluorescence imaging, the mouse was sacrificed, and five major organs (liver, lung, spleen, kidney, and heart) and tumor were excised and imaged ex vivo with an IVIS spectrum imaging system. The excised organs and tumor were washed three times using PBS and fixed using 4% formaldehyde, followed by immediate fluorescence imaging using the IVIS spectrum imaging system. All in vivo and ex vivo imaging measurements were performed using exposure times in the range of 0.5–10 s using an imaging system equipped with a 150 W Tungsten EKE illumination source (400–1500 nm wavelength range). Ex vivo fluorescence intensities were also presented as means ± standard deviation (*n* = 4).

## Supporting information

As a service to our authors and readers, this journal provides supporting information supplied by the authors. Such materials are peer reviewed and may be re‐organized for online delivery, but are not copy‐edited or typeset. Technical support issues arising from supporting information (other than missing files) should be addressed to the authors.

Supplementary
