# Supplementary material for: Genetic Assembly of Double‐Layered Fluorescent Protein Nanoparticles for Cancer Targeting and Imaging
Source: Adv Sci (Weinh). 2017 Feb 17;4(5):1600471. doi: 10.1002/advs.201600471 (PMC5441503; doi:10.1002/advs.201600471)
Supplement: Supplementary file 1 — Supplementary [file ADVS-4-na-s001.pdf]

Copyright WILEY-VCH Verlag GmbH & Co. KGaA, 69469 Weinheim, Germany, 2017.

ADVANCED SCIENCE
Open Access

Supporting Information

for *Adv. Sci.,* DOI: 10.1002/advs.201600471

Genetic Assembly of Double-Layered Fluorescent Protein Nanoparticles for Cancer Targeting and Imaging

Seong-Eun Kim, Sung Duk Jo, Koo Chul Kwon, You-Yeon Won, and Jeewon Lee**

Copyright WILEY-VCH Verlag GmbH & Co. KGaA, 69469 Weinheim, Germany, 2016.

Supporting Information

Genetic Assembly of Double-Layered Fluorescent Protein Nanoparticles for Cancer Targeting and Imaging

Seong-Eun Kim[a, b], *Sung Duk Jo*[c], *Koo Chul Kwon*[a], *You-Yeon Won*[b, c*], *Jeewon Lee*[a*]

a. Department of Chemical and Biological Engineering, Korea University, Seoul 02841, Republic of Korea
b. School of Chemical Engineering, and Purdue University Center for Cancer Research, Purdue University, West Lafayette, IN 47906-2100, U.S.A.
c. Center for Theragnosis, Korea Institute of Science and Technology, Seoul 02792, Republic of Korea

*Correspondence and requests for materials should be addressed to J.L. (leejw@korea.ac.kr) and Y.-Y.W. (yywon@ecn.purdue.edu).

Supplementary Figures

Figure S1

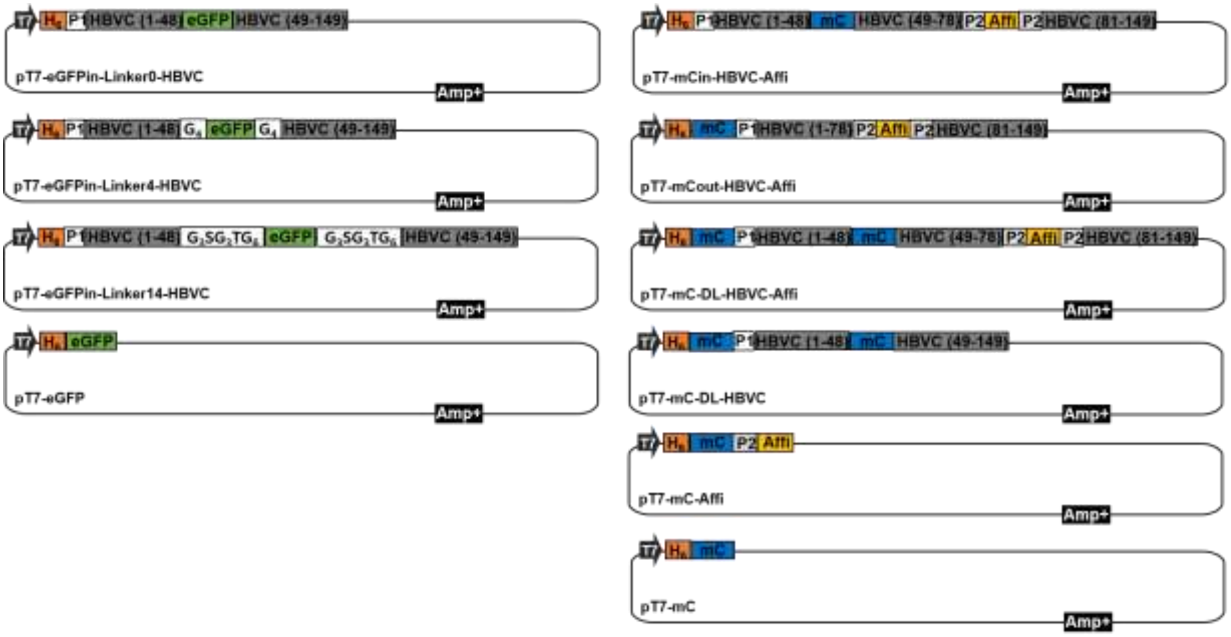

Figure S1. Plasmid vectors used for the synthesis of fluorescently engineered HBV capsids and fluorescent proteins. "Affi" represents a tandem repeat of affibody peptide. "P1" and "P2" represent the peptide sequences, MASSLRQILDSQKMEWRSNAGGSG$_3$SG$_3$TG$_6$Y$_6$ and G$_3$SG$_3$TG$_3$SG$_3$, respectively, which were used to sterically well expose the H$_6$ and affibody peptides, respectively, on the outer surface of engineered HBV capsids.

Figure S2

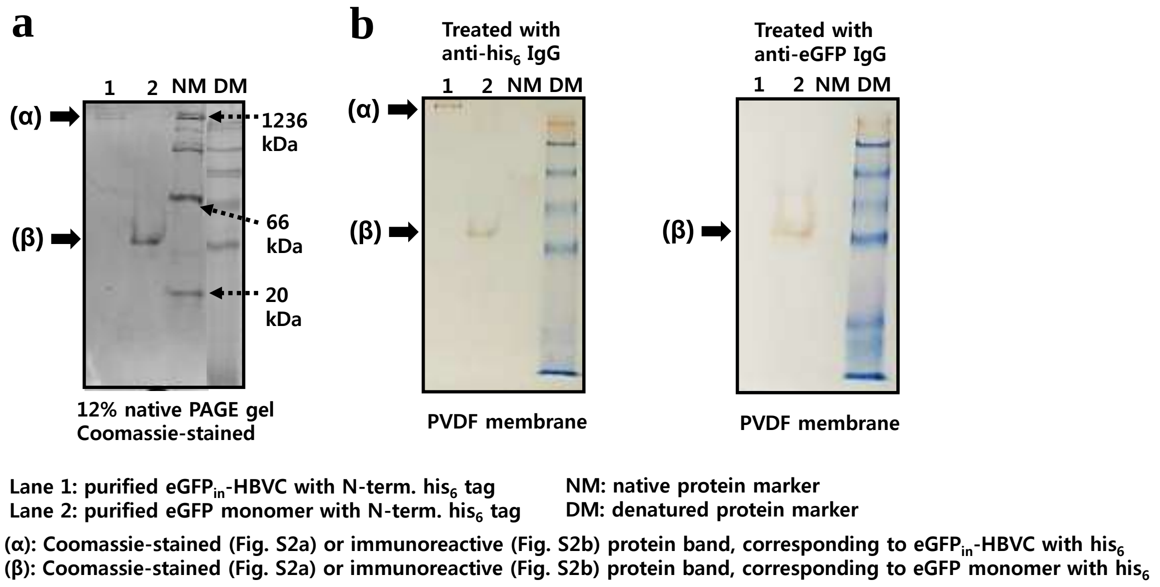

Figure S2. Analysis of native PAGE (**a**) and subsequent Western blot (**b**) of purified $eGFP_{in}$-HBVC and eGFP monomer, both of which contain his_6 tag. For Western blot analysis, anti-his_6 and anti-eGFP IgG antibodies were used as primary antibodies. The molecular weight of $eGFP_{in}$-HBVC (containing 240 subunits) is estimated to be 11,830 kDa. NM in native PAGE gel was used to confirm the size of protein bands of $eGFP_{in}$-HBVC and eGFP monomer, although is not transferrable to PVDF membrane. DM was used to show that all the proteins in native PAGE gel were completely transferred to PVDF membrane.

Figure S3

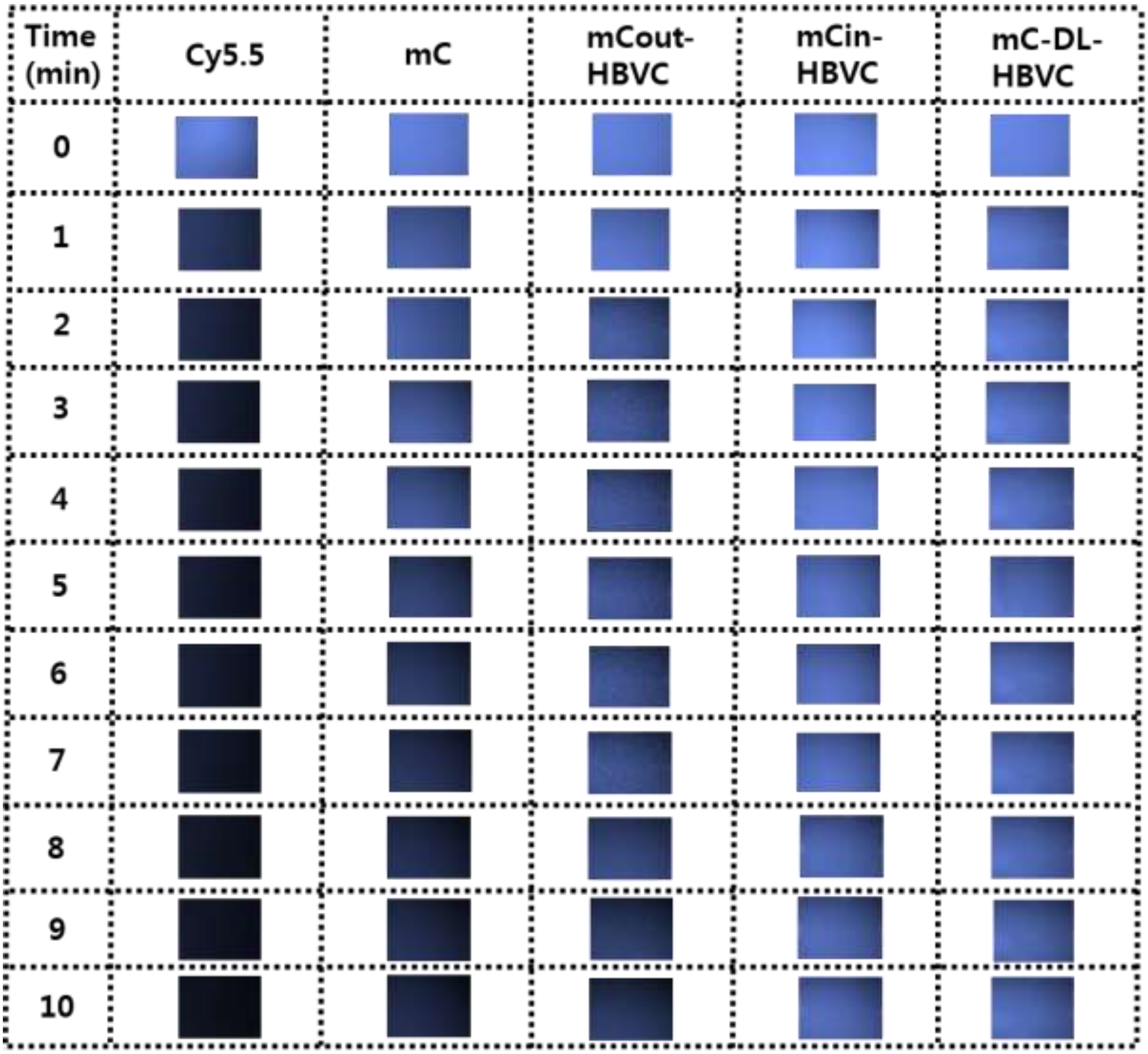

Figure S3. Fluorescence images correponding to the results of photostability analysis (Figure 2d) of Cy5.5, mC, mC_{out}-HBVC, mC_{in}-HBVC and mC-DL-HBVC.

Figure S4

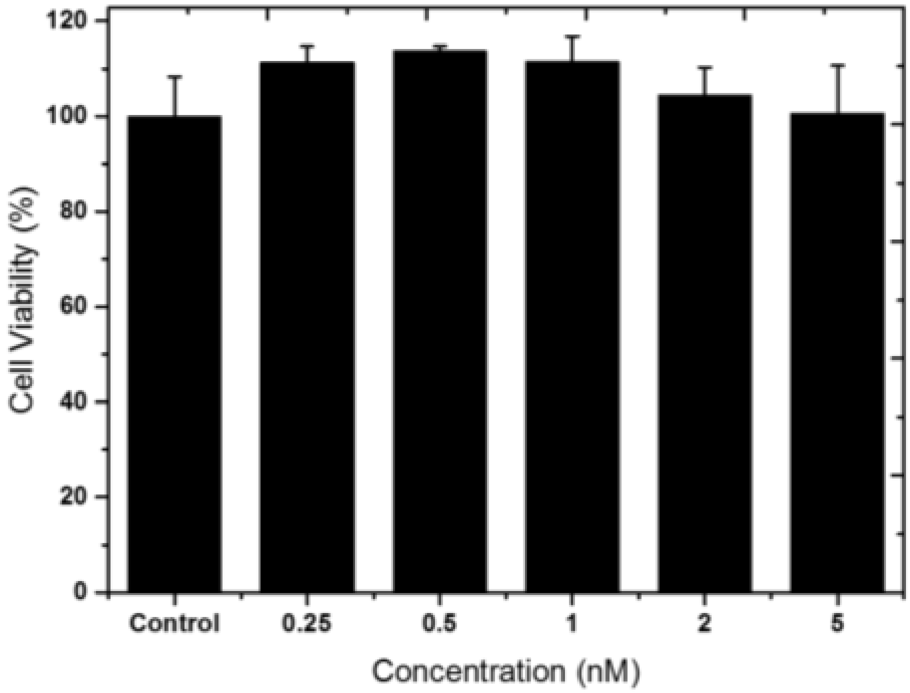

Figure S4. Viabilities of MDA-MB-468 cells treated with mC-DL-HBVC at different concentrations, measured by CCK-8 assays. Values were presented as means of triplicates with standard deviations shown as error bars.

Supplementary Tables

Table S1. Caculation of mean distance (r) between two adjacent fluorophores of eGFPs encapsulated inside the $eGFP_{in}$-HBVC

Radius (nm)* of sphere, on the surface of which fluorophores of eGFP are located	Assembly symmetry of T=3			Assembly symmetry of T=4		
	R_a = 6.5 (n = 14)	R_b = 9.3 (n = 4)	R_c = 10.4 (n = 0)	R_a = 8.0 (n = 14)	R_b = 10.8 (n = 4)	R_c = 11.9 (n = 0)
Area (nm^2) of the spherical surface where the fluorophores are located ($=S_{(a/b/c)}=4\pi R_{(a/b/c)}{}^2$)	527.7	1082.2	1359.2	800.2	1460.3	1779.5
Area (nm^2) occupied by each fluorophore (= $S_{(a/b/c)}$/number of capsid subunits)	2.9	6.0	7.6	3.3	6.1	7.4
Mean distance (nm) between two adjacent fluorophores (r) (=2 $\sqrt{(\frac{S_{(a/b/c)}}{number\ of\ subunits}) \times \frac{1}{\pi}}$)	1.9	2.8	3.1	2.1	2.8	3.1

* R_a, R_b, or R_c (nm) (see Scheme 1) is calculated by subtracting δ from inner radius of HBV capsid (12.5 nm for T = 3 symmetry; 14 nm for T = 4), where δ = length of linker peptide (3.9 nm for n = 14; 1.1 nm for n = 4, 0 nm for n = 0) + eGFP height/2 (2.1 nm). The length of linker peptide was estimated assuming that the length of one amino acid unit ($C\text{-}N\text{-}C_{\alpha}$) is 0.28 nm.
